# The salivary secretome of the biting midge, *Culicoides sonorensis*

**DOI:** 10.7717/peerj.426

**Published:** 2014-06-05

**Authors:** Christopher J. Lehiy, Barbara S. Drolet

**Affiliations:** United States Department of Agriculture, Agricultural Research Service, Arthropod-Borne Animal Diseases Research Unit, Manhattan, KS, USA

**Keywords:** Saliva, *Culicoides sonorensis*, Salivary proteins

## Abstract

*Culicoides* biting midges (Diptera: Ceratopogonidae) are hematophagous insects with over 1400 species distributed throughout the world. Many of these species are of particular agricultural importance as primary vectors of bluetongue and Schmallenberg viruses, yet little is known about *Culicoides* genomics and proteomics. Detailed studies of members from other blood-feeding Dipteran families, including those of mosquito (Culicidae) and black fly (Simuliidae), have shown that protein components within the insect’s saliva facilitate the blood feeding process. To determine the protein components in *Culicoides sonorensis* midges, secreted saliva was collected for peptide sequencing by tandem mass spectrometry. Forty-five secreted proteins were identified, including members of the D7 odorant binding protein family, Kunitz-like serine protease inhibitors, maltase, trypsin, and six novel proteins unique to *C. sonorensis*. Identifying the complex myriad of proteins in saliva from blood-feeding Dipteran species is critical for understanding their role in blood feeding, arbovirus transmission, and possibly the resulting disease pathogenesis.

## Introduction

*Culicoides* biting midges (Diptera: Ceratopogonidae) are hematophagous insects widely distributed throughout the world with over 1400 distinct species currently identified (Borkent & Wirth, 1997). Previous reports have established these midges as important vectors for a number of animal-associated pathogens including bluetongue virus (BTV) (Foster, Jones & McCrory, 1963), epizootic hemorrhagic disease virus (EHDV) (Foster et al., 1977), vesicular stomatitis virus (VSV) (Drolet et al., 2005), Schmallenberg virus (SBV) (Lehmann et al., 2012), and African horse sickness virus (AHSV) (Mellor, Boorman & Baylis, 2000), as well as the transmission of Oropouche virus which causes acute disease in humans (Mourao et al., 2009). In addition to arboviral transmission, *Culicoides* saliva introduced during blood feeding has been linked to the development of painful dermal IgE-mediated hypersensitivity responses (sweet itch) in Icelandic horses and Awassi breed sheep (Anderson, Belton & Kleider, 1991; Yeruham, Braverman & Perl, 2000).

In the United States, *Culicoides sonorensis Wirth and Jones* (*C. sonorensis*), formerly known as *Culicoides variipennis sonorensis*, is one of the most abundant midge species with a dynamic range extending from the coastal states of Florida to California (Holbrook et al., 2000). Similar to other midge species, adult *C. sonorensis* primarily nourish themselves with plant nectar with females requiring a blood meal for the maturation of fertilized eggs prior to ovipositing. Both domesticated cattle and sheep are the primary blood feeding sources for midges, although *C. sonorensis* females are also opportunistic feeders targeting a wide range of wildlife including white-tail deer, rabbits and birds (Mullens & Dada, 1992; Tempelis & Nelson, 1971). Critical to this ability to feed from multiple sources is having a salivary output replete with an assortment of sugar digestion enzymes, as well as a wide variety of proteins designed to facilitate blood feeding from multiple hosts.

Evolutionarily, mammals have co-evolved with hematophagous arthropods to develop obstacles for minimizing blood loss associated with insect feeding (Ribeiro, 1995). These obstacles include platelet aggregation, vasoconstriction, and blood coagulation, all of which are dependent upon cell signaling to be effective (Andrade et al., 2005). The process begins when damage to skin tissue during the feeding process initiates a cellular cascade leading to the formation of blood clots restricting blood flow to the site (Fontaine et al., 2011). Proteins present in the saliva of hematophagous insects actively work at inhibiting this process by sequestering and inactivating the signaling components or by providing a potent counter-signal, thereby reversing their effects. For pool feeders, such as *C. sonorensis*, one countermeasure is the formation of a feeding hematoma that provides a reservoir of blood to be extracted quickly and efficiently by multiple insects.

In addition to facilitating blood feeding, protein components in the saliva of hematophagous insects have been linked to the efficient transmission of a number of arboviruses to mammalian hosts, including West Nile virus (Schneider et al., 2006), Sindbis virus (Schneider et al., 2004), Dengue fever virus (Espada-Murao & Morita, 2011), and VSV (Limesand et al., 2000). It is believed that this efficiency is the by-product of the immunomodulatory effects of salivary components involved in blood feeding, although more work is needed to fully understand the mechanisms involved. Interestingly, a recent report involving *C. sonorensis* saliva has suggested that protein components may also be involved in the efficient transmission of BTV from an infected host into the midge (Darpel et al., 2011), but it is unclear at this time how it would affect the reciprocal transmission from an infected midge into a naïve host since the infectivity of mammalian cell cultures was reduced 2–6 fold.

A *Culicoides* salivary gland EST library containing full or partial length sequences from gland RNA was constructed in an effort to identify probable components of *Culicoides* saliva (Campbell et al., 2005). Approximately 20% (150) of the transcripts encoded a signal peptide, indicating these were potentially secreted in saliva. Further proteome analysis of *C. sonorensis* saliva by one-dimensional gel electrophoresis analysis suggested that the number of proteins produced may actually be much less than that predicted by the transcriptome analysis (Darpel et al., 2011). In line with this, other studies comparing the proteome of various organisms including the fruit fly *Drosophila melanogaster* with either their genomes or their transcriptomes have found significant differences in numbers of predicted proteins, which is often attributed to environmental factors, as well as various methods of post-translational control (Diz, Martinez-Fernandez & Rolan-Alvarez, 2012; Schrimpf et al., 2009).

To specifically analyze the protein components of secreted *C. sonorensis* salivary proteins (secretome), without contamination of salivary gland-derived proteins, a previously reported artificial membrane collection method (Langner et al., 2007) was used to recover saliva from 3 to 17 day old adult midges over the course of four months. Protein components of the saliva were identified using a Tandem Mass Spectroscopy (MS/MS) approach. Possible functional roles of identified proteins are discussed.

## Materials and Methods

### *Culicoides* saliva collection

Saliva was collected from the Arthropod-Borne Animal Diseases Research Unit (ABADRU)-maintained *C. sonorensis* colony (Jones, 1960), over a four month period using an artificial membrane feeder system as previously described (Langner et al., 2007). Briefly, midges were maintained during collection on a 10% sucrose diet. Saliva was collected onto Millipore 0.22 µm hydrophilic membrane discs (Millipore, Billerica, MA) from 3 to 17 day old adult female midges daily. The discs were placed into phosphate buffered saline (PBS) pH 7.4 supplemented with 1 mM 3-[(3-Cholamidopropyl) dimethylammonio]-1-propanesulfonate (CHAPS; Sigma Aldrich, St. Louis, MO) agitated via vortex for 5 min and stored at 4 °C for up to one week. CHAPS-PBS-saliva solution collected for the period was concentrated using an Amicon Ultra-15 Centrifugal Filter with Ultracel-3 membrane according to manufactures guidelines (Millipore) and then stored at −80 °C until use. Protein concentrations were estimated using a nanophotometer (Implen, Westlake Village, CA).

### SDS polyacrylamide Gel Electrophoresis (PAGE)

To maximize protein loading on the gel, 125 µg of total salivary protein was concentrated by precipitation using the ReadyPrep 2D Cleanup kit (Biorad), resuspended in 50 µL of Laemmli sample buffer supplemented with 2% *β*-mercaptoethanol (Biorad) and heated at 95 °C for 5 min. Proteins from the pooled and concentrated samples, along with Precision Plus Protein Kaleidoscope standards (Biorad, Hercules, CA), were separated on a 4–20% Mini-PROTEAN^®^ TGX™ Precast Gel (Biorad, Hercules, CA) for subsequent peptide sequencing by tandem mass spectrometry (MS/MS) analysis. After electrophoresis, the gel was rinsed with three changes of Nanopure water, stained for 20 min with Bio-safe™ Coomassie (Biorad) and destained with three changes of Nanopure water (Biorad). The gel lane was carefully cut into seven sections, placed into Eppendorf tubes and rinsed twice with 1 mL of 5% acetonitrile. The tubes were stored at −20 °C prior to shipment to Harvard University’s Mass Spectroscopy and Proteomics Resource Core facility. For reference, another lane was stained with the Bio-Rad Silver stain kit (Biorad) following the manufacturer’s protocol.

### MS/MS sequencing

Sequencing was performed at the Harvard University Mass Spectrometry and Proteomics Resource Laboratory (Cambridge, MA) using a microcapillary reverse phase high pressure liquid chromatography (RP-HPLC) unit coupled to an LTQ-Orbitrap XL mass spectrometer (Thermo-Scientific, Waltham, MA). The individual spectra generated were then correlated to known protein sequences using the SEQUEST algorithm and proprietary software (Chittum et al., 1998; Eng, Mccormack & Yates, 1994).

### Database construction

The reference database used for spectral mapping and annotation was comprised of over 20,000 protein sequences downloaded from the National Center for Biotechnology Information (NCBI) depository. In addition to all the available known or predicted *Culicoides* proteins, known and/or predicted salivary proteins from black fly species *Simulium nigrimanum and Simulium vittatum*; hard and soft bodied tick species *Amblyomma americanum*, *Amblyomma maculatum, Amblyomma variegatum, Argas monolakensis, Dermacentor andersoni, Haemaphysalis longicornis, Hyalomma asiaticum, Hyalomma dromedarii, Hyalomma marginatum rufipes, Ixodes pacificus, Ixodes persulcatus, Ixodes ricinus, Ixodes scapularis, Ornithodoros coriaceus, Ornithodoros parkeri, Ornithodoros savignyi, Rhipicephalus appendiculatus, Rhipicephalus haemaphysaloides, Rhipicephalus microplus, Rhipicephalus pulchellus, and Rhipicephalus sanguineus;* sandfly species *Lutzomyia ayacuchensis, Lutzomyia longipalpis, Phlebotomus arabicus, Phlebotomus argentipes, Phlebotomus ariasi, Phlebotomus papatasi and Phlebotomus perniciosus;* and mosquito species *Aedes aegypti, Aedes albopictus, Anopheles funestus, Anopheles gambiae, Anopheles stephensi, Culex quinquefasciatus,* and *Culex tarsalis* were included. Contaminant proteins were identified using a common contaminant database provided by the Harvard Mass Spectrometry and Proteomics Resource Laboratory, and these were removed from the final analysis.

### Amino acid sequence and conserved domain analysis

*C. sonorensis* sequences deduced from MS/MS spectra were analyzed using the Domain Enhanced Lookup Time Accelerated Basic Local Alignment Search Tool (Delta-BLAST) available from the NCBI. Results were limited with an Expected (E) value cutoff of 0.01 and a low complexity filter. Multiple amino acid sequence alignments were assembled with MEGA5 software utilizing the high accuracy and high throughput (MUSCLE) algorithm (Edgar, 2004). Phylogenetic inference under maximum likelihood for the D7 family of related proteins was computed using MEGA5 software based on the JTT matrix based model (Tamura et al., 2011).

## Results and Discussion

### *Culicoides* saliva secretome proteins identified

**Figure 1:**
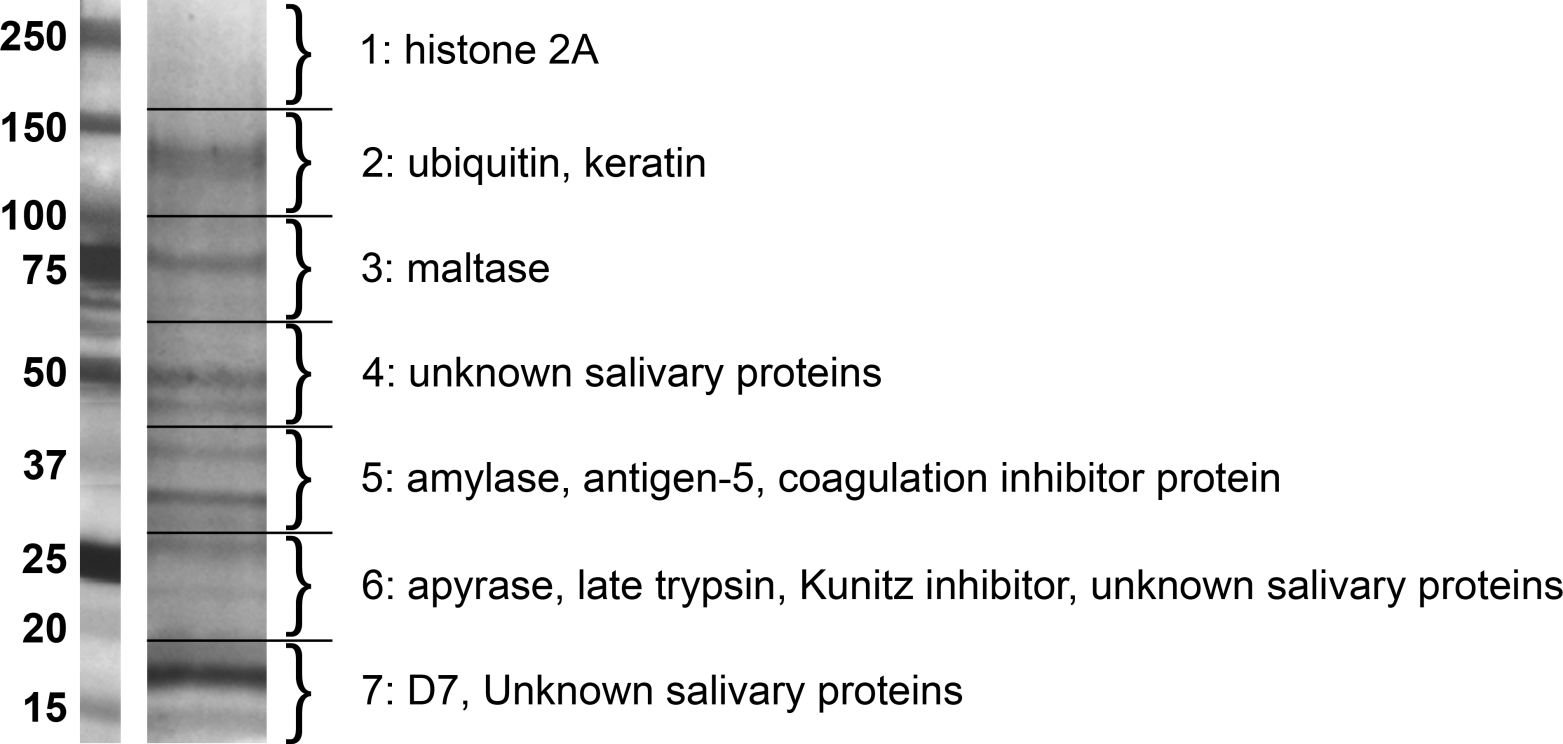
Gel separation of *Culicoides sonorensis* salivary proteins. Silver stained gel with the most abundant proteins in each segment noted. A total of 45 proteins were identified by tandem mass spectroscopy from the seven segments.

Two independent sequencing events of the seven gel fragments (Fig. 1) produced 9313 *Culicoides sonorensis* peptides that aligned to the reference database by the Harvard Mass Spectrometry and Proteomics Resource Laboratory. A subset of 664 peptides were omitted, as they correlated to proteins without a defined signal sequence including several 40S ribosomal proteins, alpha-tubulin, and heat shock protein 60 and were omitted. The remaining 8649 peptides mapped to 45 *C. sonorensis* partial and full length protein sequences. The gross number of peptide sequences, weighted against protein size to remove small protein bias, provides the relative abundance for each salivary protein which is presented in Table 1.

**Table 1:** *Culicoides sonorensis* secretome. Secretome assembled from the two MS/MS sequencing reports. The order of the sequences is according to their relative abundance. Homology searches were done using Delta-BLAST tools and the non-redundant protein database from NCBI.

Protein accession name	% coverage	Homologs/orthologs	% I/S
gi∣51557681 Maltase	73.9%	Cul n 8 allergen [*C. nubeculosus*: gI∣300078745]	92/97
gi∣51557677 Late Trypsin	70.1%	Trypsin [*C. nubeculosus*: gI∣221768504]	64/81
gi∣51557760/62 D-7 related	84.3/30.8%	D7-like salivary protein [*C. nubeculosus*: gI∣221768675]	60/76
gi∣51557806 Unknown	77.1%	Cul n 4 allergen [*C. nubeculosus*: gI∣300078737]	89/95
gi∣51557786 Protease inhibitor	82.0%	Putative salivary protease inhibitor [*C. nubeculosus*: gI∣221768811]	74/84
gi∣51557689 D-7 related	88.4%	Cul n 09 allergen [*C. nubeculosus*: gI∣300078747]	82/87
gi∣51557816 Unknown	85.0%	Putative IgE binding protein [*C. nubeculosus*]: GI:313482949	41/59
gi∣51557713 Unknown	73.4%	Putative salivary protease inhibitor [*C. nubeculosus*: gI∣221768800]	74/85
gi∣51557796/98 D-7 related	81.9/50.3%	D7-like salivary protein [*C. nubeculosus*: gI∣221768844]	63/74
gi∣51557709 Unknown	82.8%	Cul n 06 allergen [*C. nubeculosus*: gl—300078741]	82/91
gi∣51557728/19 Unknown	74.9/73.9%	Cul n 10 allergen [*C. nubeculosus*: gI∣300078749]	77/89
gi∣51557828 Kunitz-like inhibitor	84.4%	Tissue factor pathway inhibitor [*C. nubeculosus*: gI∣221768840]	32/47
gi∣51557812 D-7 related	78.3%	D7-like salivary protein [*C. nubeculosus*: gI∣221768758]	78/91
gi∣51557661 Antigen-5 related	79.8%	Salivary antigen-5 related protein AG5-1 [*A. albopictus*: gI∣56417468]	43/57
gi∣51557764 Unknown	87.3%	Novel	
gi∣51557820/18 Unknown	72.8/21.5%	Secreted salivary protein [*C. nubeculosus*: gI∣221768554]	84/91
gi∣51557752 Unknown	80.1%	Cul n 4 allergen [*C. nubeculosus*: gI∣300078737]	37/58
gi∣51557800/02/04 Unknown	79.6/76.6/32.1%	Secreted salivary protein [*C. nubeculosus*: gI∣221768768]	69/81
gi∣51557826 Coagulation inhibitor	88.6%	Apyrase [*Tabanus yao*: gI∣374110471]	53/67
gi∣51557693 D-7 related	74.8%	D7-like salivary protein [*C. nubeculosus*: gI∣221768569]	70/82
gi∣51557726 Unknown	50.0%	Secreted salivary protein [*C. nubeculosus*: gI∣221768825]	85/91
gi∣51557717 Unknown	76.3%	Novel	
gi∣51557822/24/7701 Unknown	46.7/6.4/57.4%	Cul n 5 allergen [*C. nubeculosus*: gI∣300078739]	78/85
gi∣51557792 Unknown	40.1%	Putative salivary protease inhibitor [*C. nubeculosus*: gI∣221768544]	68/8
gi∣51557675 Hyalurono-glucosaminidase	58.8%	Cul n 2 allergen [*C. nubeculosus*: gI∣300078733]	94/98
gi∣51557665 Amylase	54.2%	alpha amylase [*Musca domestica*: gI∣139478961]	58/74
gi∣51557772 Unknown	59.1%	Secreted salivary protein [*C. nubeculosus*: gI∣221768554]	74/84
gi∣51557776 Kunitz-like inhibitor	74.0%	Putative salivary protease inhibitor [*C. nubeculosus*: gI∣221768578]	31/47
gi∣51557766 Unknown	54.8%	Novel	
gi∣51557810 D-7 related	44.6%	D7-like salivary protein [*C. nubeculosus*: gi∣221768758]	76/90
gi∣51557663 Antigen-5 related	44.9%	Antigen 5-related salivary protein [*C. nubeculosus*: gi∣221768626]	86/94
gi∣51557695 Unknown	45.0%	Secreted salivary protein [*C. nubeculosus*: gi∣221768524]	38/59
gi∣51557830 Tissue factor pathway inhibitor	38.0%	Tissue factor pathway inhibitor [*C. nubeculosus*: gi∣221768840]	76/86
gi∣51557705 Unknown	65.6%	Novel	
gi∣51557814 Unknown	58.9%	Secreted salivary protein [*C. nubeculosus*: gI∣221768786]	41/60
gi∣51557715 Unknown	32.1%	Cul n 4 allergen [*C. nubeculosus*: gi∣300078737]	44/65
gi∣51557742 Unknown	42.3%	Novel	
gi∣51557778 Kunitz-like inhibitor	26.6%	Putative salivary protease inhibitor [*C. nubeculosus*: gi∣221768483]	28/46
gi∣51557770 Unknown	35.1%	Cul n 7 allergen [*C. nubeculosus*: gi∣300078743]	77/85
gi∣51557818 Unknown	21.5%	Secreted salivary protein [*C. nubeculosus*: gI∣221768554]	84/91
gi∣51557734 Unknown	40.6%	Novel	
gi∣51557691 Unknown	16.9%	Laminin-like secreted protein [*C. nubeculosus*: gI∣221768562]	82/94
gi∣51557724 D-7 related	23.0%	D7-like salivary protein [*C. nubeculosus*: gi∣221768533]	30/50
gi∣51557738 Endopeptidase	23.0%	Putative cysteine endopeptidase [*C. nubeculosus*: gI∣221768705]	70/84
gi∣56199478 Unknown	13.7%	Hypothetical protein [*Aedes aegypti*; gI∣157124809 ]	49/63

**Notes.**

I Identity S Similarity

### Conserved domains of *Culicoides* salivary secretome proteins

**Figure 2:**
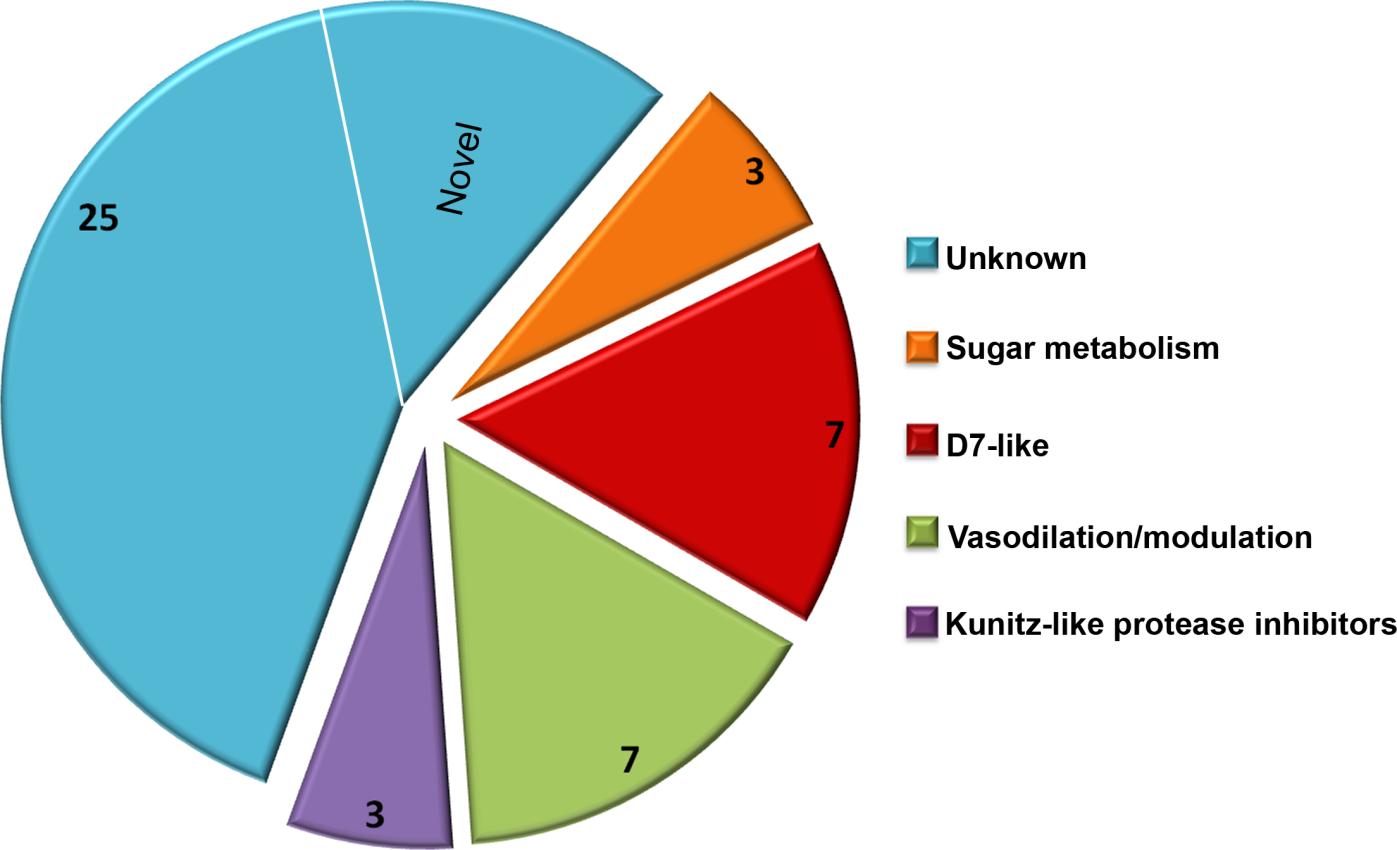
*Culicoides* secretome functional identification. Based on homology searches with previously identified insect proteins, the 45 proteins identified were placed into six broad function categories. (1) *Unknown*. There were 25 proteins of unknown function with homologs found in other *Culicoides* species, most often *C. nubeculosus* indigenous to Europe; (2) *Sugar metabolism*. Maltase and amylase, together constituted 28% of the total sequences identified. While primarily involved in the digestion of complex sugars, maltase may also be involved in protecting the insect from heme during red blood cell digestion; (3) *D7 proteins*. Members of the pheromone-general odorant binding protein superfamily, these proteins have a well-defined hydrophobic pocket held in place through cysteine bonds. Previous studies with D7-like proteins from mosquitoes have shown their ability to bind to various biogenic amines including histamine and epinephrine; (4) *Vasodilation/modulation*. Proteins such as tissue factor pathway inhibitor directly inhibit the host’s ability to restrict blood flow; and (5) *Kunitz-like protease inhibitors*. These, along with the vasodilation/modulation proteins allow for efficient blood feeding from diverse hosts.

Due to a large number of proteins annotated as uncharacterized after a screen of the reference database, Delta-BLAST was used to identify potential orthologs or previously unidentified Structural Classification of Proteins (SCOP) domains. The majority of the secretome proteins (25 of 45) had no discernible domains under the search criteria used, and these were classified as unknown salivary proteins in Table 1. Eight distinct SCOP domains were identified among the remaining 20 proteins. Four of these domains are found in several of the most abundant *C. sonorensis* salivary proteins including alpha-glucosidase found in maltase, trypsin-like serine protease found in late trypsin, pheromone binding protein-general odorant binding protein (PBP-GOBP) found in the D7-family, and Kunitz-bovine pancreatic trypsin inhibitor (BPTI) found in the Kunitz-like serine protease inhibitor proteins. Other conserved structural elements identified in this search were the 5’nucleotidase domain found in the coagulation inhibitor-like protein, the hyaluronidase domain in hyaluronoglucosaminidase, the alpha-amylase domain found in amylase, and the sterol carrier protein (SCP)-like domain found in the two antigen-5 related proteins. Based on these homology searches with previously identified insect proteins, the 45 secreted proteins of *Culicoides* saliva were placed into five broad categories (Fig. 2): sugar metabolism, D7 proteins, vasodilation/modulation, Kunitz-like protease inhibitors and unknowns.

As expected, the Delta BLAST search of the 45 *C. sonorensis* protein sequences also resulted in the identification of a number of potential homologs present in *C. nubeculosus*, a *Culicoides* spp. native to Europe. The amino acid identity between these *Culicoides* sequences ranged from an average of 31% for the Kunitz-like serine protease inhibitors to 93% for the maltase proteins. The Delta BLAST search also revealed several closely related orthologs to *C. sonorensis* salivary proteins. These include the antigen-5 related protein to AG5-1 from the mosquito *Aedes albopictus* (43% identity/57% similarity); the coagulation inhibitor-like protein to the apyrase protein from the horsefly, *Tabanus yao* (53% identity/64% similarity); and the amylase protein to the alpha amylase from the common housefly, *Musca domestica* (58% identity/74% similarity). Interestingly, six proteins within the *C. sonorensis* secretome had no discernible SCOP domains or closely related proteins in other Dipterans including *C. nubeculosus*, and these were identified as novel.

The salivary secretome of *C. sonorensis* midges encompasses at least 45 distinct proteins with a wide distribution of relative abundance. This number is significantly lower than the one predicted from the previous transcriptome analysis of the salivary glands (Campbell et al., 2005), although at this time we cannot rule out the possibility that other proteins are present but below the level of detection used here. Interestingly, the ten most abundant proteins in the saliva accounted for nearly 83% of the total sequences, perhaps suggesting the remaining 35 proteins are functionally redundant or active at very low concentrations. Delta-BLAST and SCOP domain searches of the 45 protein sequences identified 25 with no clearly related homology to previously characterized proteins and remain classified as unknown salivary proteins. The remaining 20 sequences have significant homology to several functionally characterized proteins useful to hematophagous arthropods.

### *Culicoides sonorensis* maltase

**Figure 3:**
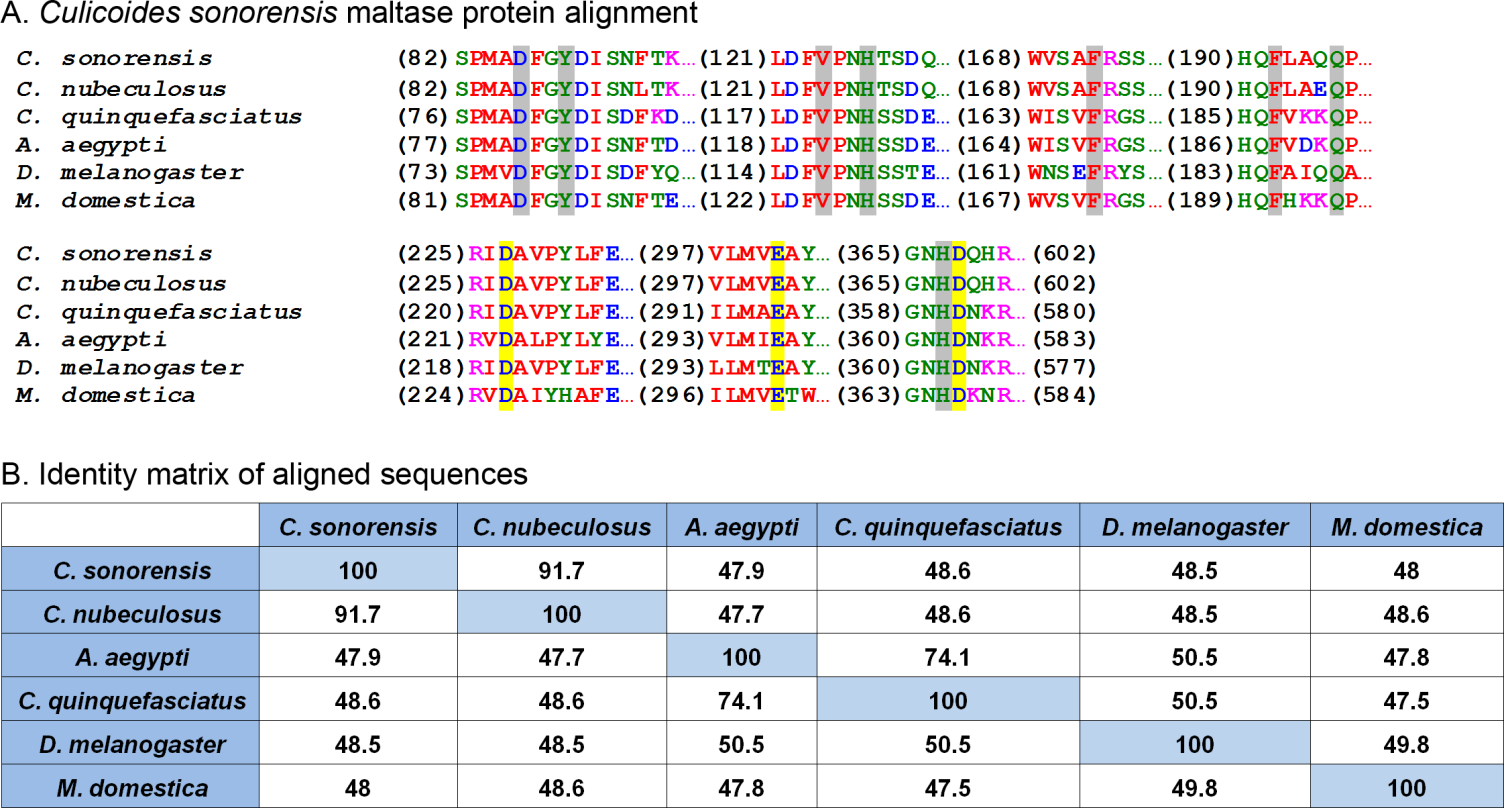
*Culicoides sonorensis* maltase protein. (A) Alignment of the maltase protein with putatively identified maltase proteins from a number of insect species. Grey highlighted residues correspond to the conserved active site residues responsible for preferential binding to the specific carbohydrate prior to hydrolysis. The invariant catalytic triad (Asp-Glu-Asp) responsible for the nucleophilic exchange is highlighted in yellow. (B) Amino acid identity matrix of the aligned sequences. As expected, the closely related *C. nubeculosus* shows the highest degree of conservation with *C. sonorensis* although all the proteins highlighted here have >47% identity.

One of the most abundant proteins identified in the secretome shares homology with a wide assortment of maltase enzymes found in the saliva of mosquitoes and other blood feeding insects, as well as the blood and urine of mammals. These enzymes, classified as type I alpha glucosidases, are differentiated from other alpha-glucosidases based on the conservation of four discrete amino acid regions found in their (*β*/*α*)_8_ barrel structure (Fig. 3). These regions are important for substrate binding as well as maintaining the catalytic triad (Asp-Glu-Asp) responsible for the hydrolysis of the alpha 1-4 linkage of the disaccharide maltose (Krasikov, Karelov & Firsov, 2001). Interestingly, studies involving alpha glucosidases from mammals showed the enzyme could bind to and hydrolyze other complex sugars including soluble starch, albeit with a lower affinity compared to maltose, suggesting that maltase enzymes could potentially function independent of amylase (Chiba & Shimomura, 1978).

At the protein level, maltase from *C. sonorensis* is predicted to be a 66.8 kilodalton (kDa) protein with a theoretical isoelectric point (pI) of 4.88, similar to acidic alpha glucosidases associated with the midgut. It shares 49% identity/68% similarity with midgut associated maltase H from *Drosophila melanogaster* (gi∣41712585) and 50% identity/68% similarity with the midgut maltase from *Aedes aegypti* (gi∣108873258). The protein identity matrix of *C. sonorensis* maltase with alpha glucosidase enzymes from other species (Fig. 3B) illustrates the high degree of conservation of four discrete regions as previously identified for type I alpha glucosidases (Krasikov, Karelov & Firsov, 2001), as well as the presence of the conserved catalytic triad. All glucosidases have a greater than 47% identity with *C. sonorensis* maltase. Previous studies have established the *C. sonorensis* maltase as one of the major allergens associated with the hypersensitivity response in horses (Langner et al., 2009), but to date it has not been functionally characterized. Midgut associated maltase enzymes have not been identified in *C. sonorensis* or in the closely related *C. nubeculosus*. It is possible that the maltase produced in saliva, and ingested during feeding, functions in the lumen of the midgut to aid in sugar digestion. It should also be noted that in other hematophagous insects such as the kissing bug, *Rhodnius prolixus*, alpha glucosidases associated with the midgut are responsible for the sequestration of heme after blood meals (Mury et al., 2009). It is, therefore, possible that maltase may be serving two roles for *Culicoides* depending on the ingested meal. When nectar or sugar is the food source, maltase may be functioning to aid in the hydrolysis of maltose to glucose. Alternatively, when females ingest a blood meal, maltase may serve a protective role in sequestering digestion byproducts such as heme to protect midgut epithelial cells.

### *Culicoides sonorensis* late trypsin

**Figure 4:**
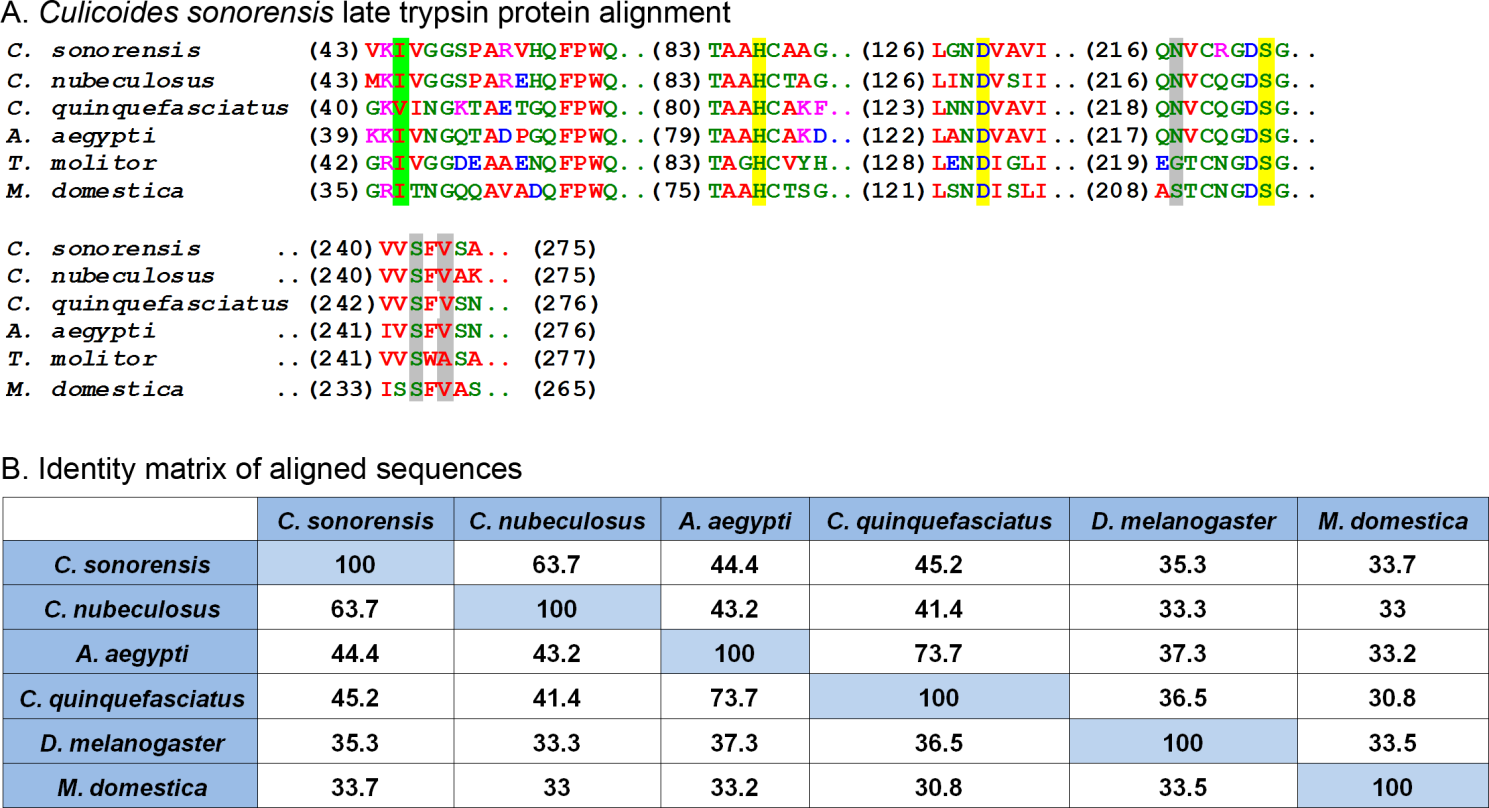
*Culicoides sonorensis* late trypsin protein. (A) Alignment of late trypsin protein with a number of putatively identified insect serine proteases. Areas of interest include the zymogen cleavage site highlighted in green, and the substrate binding sites highlighted in gray. The highly conserved serine protease catalytic triad (His-Asp-Ser) involved in the nucleophilic cleavage of the target substrate is highlighted in yellow. (B) Amino acid identity matrix of the aligned sequences. Conservation between the serine proteases outside on the requisite catalytic triad is relatively low with *C. nubeculosus* trypsin being the closest to *C. sonorensis* late trypsin at 63.7%. The variation in sequence is somewhat expected considering the high degree of substrate diversity found among serine proteases and the role protein folding plays in determining the target affinity.

Another protein in relative high abundance in the *C. sonorensis* secretome shares homology with a wide variety of insect trypsin-like serine proteases including one from the saliva of *A. aegypti* (48% identity/64% similarity) and another from *Culex quinquefasciatus* (45% identity/64% similarity) (Fig. 4). These proteases are typically stored as zymogens which are activated during the deposition of saliva upon feeding either through autolysis or through the action of a host provided protease (Meiser et al., 2010). Once cleaved, serine proteases undergo a conformational change bringing together a catalytic triad of residues (His-Ser-Asp) in an active site cleft. This triad gives specificity to the peptide cleavage site which, in the case of a serine protease, is a lysine or arginine residue. The function of serine proteases in saliva is not well understood, but it is believed they may act as a defense against orally transmitted pathogens or perhaps function as an anti-coagulant by cleaving Protein C during bloodfeeding to prevent clotting (Francischetti et al., 2009).

In *C. sonorensis*, there were 28 serine protease clusters previously identified with two, LTRYP3A and LTRYP3B, being isolated primarily in the salivary glands of adult males and females (Campbell et al., 2005). Quantitative real-time PCR analysis of LTRYP3A showed its transcript levels increased when females fed on serum compared to unfed controls and it was given the designation of late trypsin in accordance to *Drosophila* naming convention for early and late midgut trypsin proteins (Barillasmury, Noriega & Wells, 1995). The mature, active form of late trypsin is predicted to be a 24.6 kDa protein with a theoretical pI of 9. Late trypsin has been partially characterized with respect to its ability to cleave the outer capsid proteins (VP2) from BTV-1, BTV-16 and an Eastern variant of EHDV-2 (Darpel et al., 2011). Cleavage of VP2 resulted in the formation of infectious subviral particles with an approximate 10-fold increased infectivity in *C. sonorensis* derived KC cells. Interestingly, cleavage of VP2 by late trypsin resulted in a 2–6 fold loss of infectivity in baby hamster kidney (BHK) cells. This would suggest that late trypsin could enhance the transmission of BTV infection from an infected host to a susceptible *Culicoides*, but could also result in reduced transmission rates from an infected *Culicoides* to a susceptible mammalian host.

### *Culicoides sonorensis* Kunitz-type protease inhibitors

Kunitz-type protease inhibitors are a large (>1600 member), functionally diverse superfamily of proteins characterized by the presence of at least one small (<60 amino acid) disulfide rich alpha-beta (KU) domain. Perhaps the best studied example of a Kunitz-type protease inhibitor is the bovine pancreatic trypsin inhibitor (aprotinin); a 79 amino acid single KU protein which is capable of inhibiting a number of serine proteases at the nanomolar concentration (Ascenzi et al., 2003). In arthropods, there are at least 631 proteins with recognized KU domains including a significant number of anti-hemostatic proteins found in the saliva of blood feeding arthropods. These include the potent factor Xa inhibitor of Ixolaris from *Ixodes scapularis*, and the tissue factor pathway inhibitor (TFPI) from ticks, as well as the anti-thrombin peptide anophelin isolated from mosquito saliva (Francischetti, Valenzuela & Ribeiro, 1999; Mans & Neitz, 2004). Structural analysis of the anti-hemostatic factors isolated from both hard and soft bodied ticks has shown that folding patterns of the disulfide rich KU domains allows for irreversible binding to the active site clefts of factor VII/Xa or thrombin preventing their function (Corral-Rodriguez et al., 2009).

**Figure 5:**
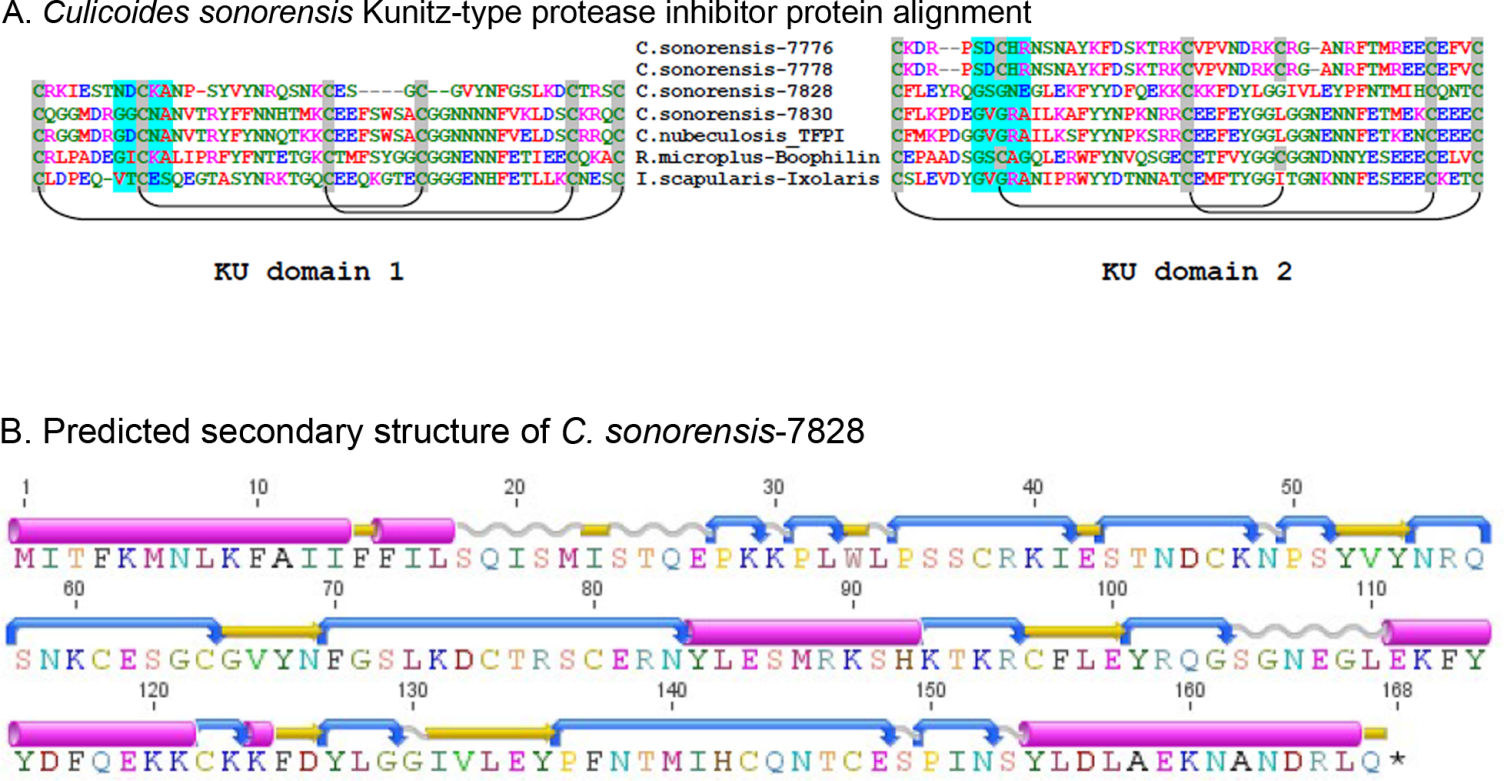
*Culicoides sonorensis* Kunitz-type protease inhibitor. (A) Sequence alignment of the Kunitz-type domains of the protease inhibitor proteins in *C. sonorensis* (unique identifiers correspond to the last four GI-accession numbers) along with several well characterized arthropod protease inhibitors. Kunitz domains are stabilized by extensive, conserved disulfide bridges with predicted linkages shown here. The potential trypsin interaction site for the domains is shaded blue. Both *C. sonorensis*-7776 and *C. sonorensis*-7778 have one predicted KU domain, but currently only partial sequences are available for each leaving open the possibility they have another defined KU-domain. (B) Predicted secondary structure of *C. sonorensis*-7828 showing the typical alpha+beta fold (blue-turn, yellow-beta strand, red-alpha helix) of the Kunitz domains. This structure allows for substrate specificity for various proteases.

In *C. sonorensis*, four proteins were identified in the saliva with at least one discernible KU domain (Fig. 5), two of which are partial sequences (gi∣51557776 and gi∣51557778) and two are full-length (gi∣51557828 and gi∣51557830). These domains are disulfide rich with four to six conserved cysteine residues within each, predicted to form three disulfide linkages (Ferre & Clote, 2005a; Ferre & Clote, 2005b). The most abundant of these proteins, the Kunitz-like protease inhibitor (gi∣51557828), appears to be highly divergent from other inhibitors sharing limited homology (42% identity/68% similarity) with protein Y43F8B.3 from *Caenorhabditis elegans*, (gi∣453232738) but little to no homology with other known Kunitz-like proteins from insects including TFPI (gi∣221768840) from *C. nubeculosus* (32% identity/47% similarity) and papilin from *A. aegypti* (28% identity/44% similarity). A conserved domain search with this Kunitz-like protease inhibitor showed that only one of the two KU domains had a potential trypsin interaction site further indicating a novel function. Conversely, TFPI from *C. sonorensis* shares 76% identity/86% similarity with TFPI from *C. nubeculosus* and 51% identity/66% similarity with a Kunitz-type inhibitor from *A. aegypti* (gi∣157126939). Furthermore, both domains in *C. sonorensis* TFPI have clear trypsin interaction sites suggesting it can interact with both factor VII and factor Xa to act as an anti-hemostatic factor.

### *Culicoides sonorensis* D7 proteins

A family of D7 proteins present in *C. sonorensis* saliva is also of interest. D7 proteins are members of the larger pheromone-general odorant binding protein (PBP-GOBP) superfamily found in arthropods. Within the D7 family, there are two major classes of odorant binding proteins (OBPs) in the saliva of hematophagous Dipterans: a short form variant which is 15–20 kDa in size containing a single odorant binding domain (OBD) and a long form which is approximately 30 kDa in size and contains two OBDs (Valenzuela et al., 2002). Many hematophagous insects appear to encode both forms, with the exception of sand flies which appear to encode only long form variants (Anderson et al., 2006; Rohousova et al., 2012). Crystal structures of a short form variant from *Anopheles gambiae*, D7r4, and a long form variant from *A. aegypti,* AeD7, showed the critical role of a set of conserved cysteine residues in forming a pocket within the OBD with an ability to bind to several biogenic amines essential for hemostasis including histamine, norepinephrine, and serotonin (Calvo et al., 2009; Mans et al., 2007). *In vitro* analysis of four short variants from *A*. *gambiae* and a long form variant from *A. aegypti* showed binding affinity to these biogenic amine compounds was sequence dependent suggesting the multiple forms of D7s were necessary to efficiently remove these compounds during feeding (Calvo et al., 2006). Additionally, a D7 homolog found in *Anopheles stephensi*, AnSt-D7L1, was found to be incapable of interacting with biogenic amines due to a change in the folding pattern of the C-terminal OBD. This new folding pattern, however, created an elongated loop that was capable of binding to the active site of thromboxane A_2_. This subtle change in the OBD allowed AnSt-D7L1 to inhibit platelet aggregation in a concentration dependent manner (Alvarenga et al., 2010).

**Figure 6:**
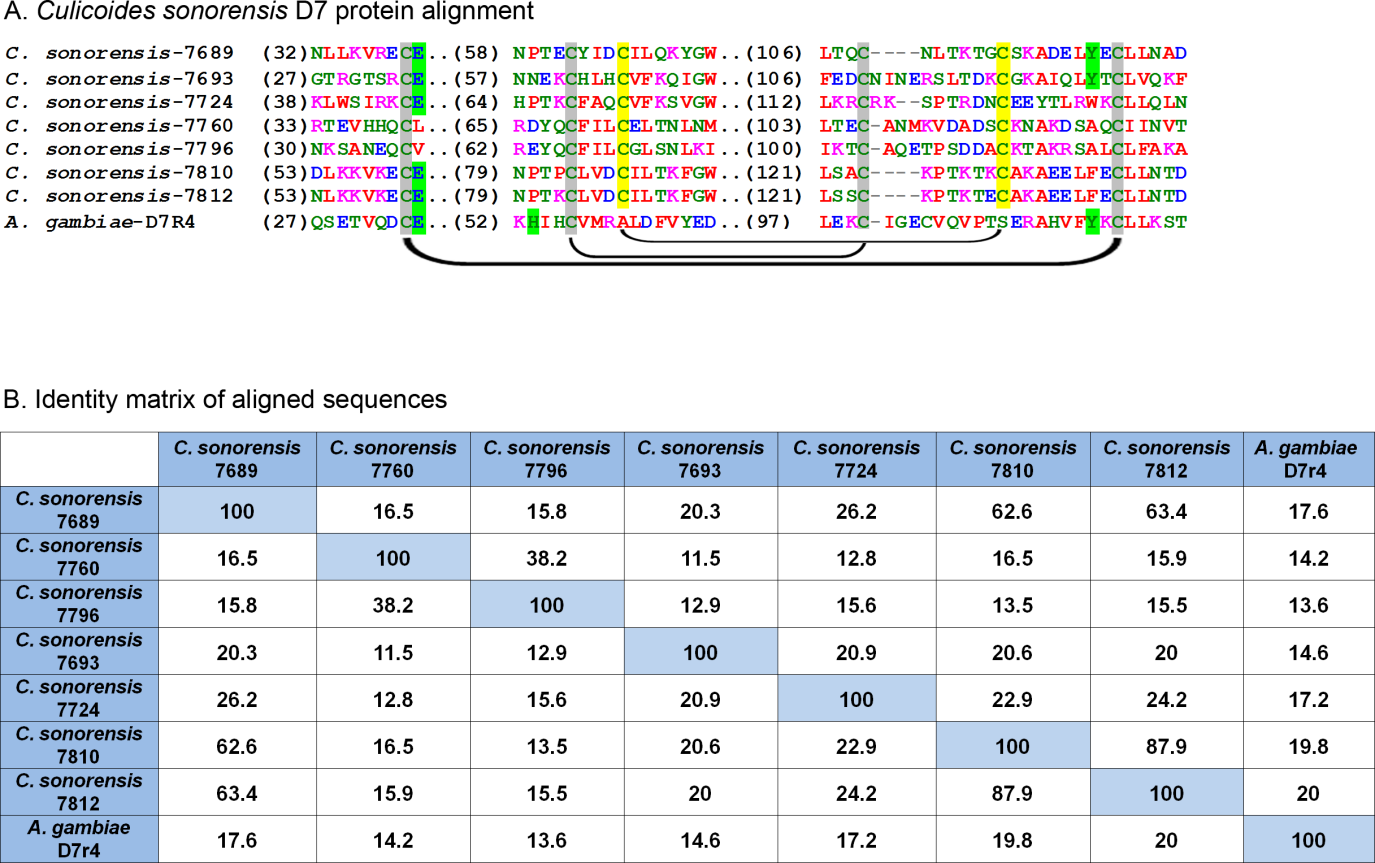
*Culicoides sonorensis* D-7 like proteins. (A) Alignment of the D7-like proteins from *C. sonorensis* (unique identifiers as the last four numbers of the accession numbers) with the *Anopheles gambiae* D7r4 short form variant. In the Anopheline variants, stabilization of the extended alpha helix segments into a binding pocket is maintained through three disulfide bridges, two of which are conserved in *Culicoides* variants (marked in grey). A third bridge, highlighted in yellow, is predicted in the *C. sonorensis* D7 proteins but absent in the D7R4 variant. The crystal structures of D7R4 complexed with the biogenic amines serotonin, tryptamine, histamine, and norepinephrine showed several key residues responsible for binding (highlighted in green) which are not completely conserved in the *Culicoides* variants. (B) While the helical structures of the *C. sonorensis* variants are largely conserved, their sequence identity is not.

**Figure 7:**
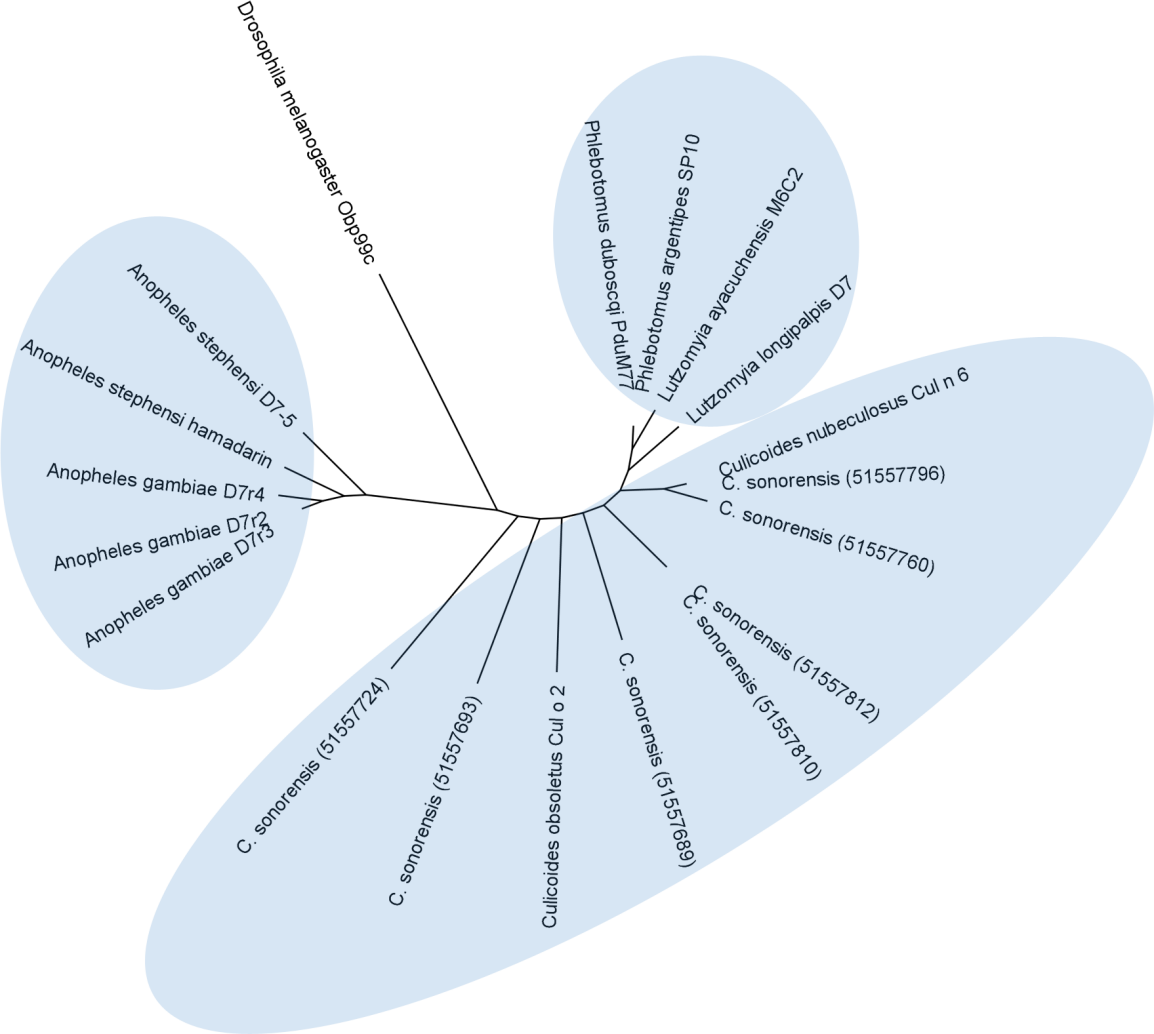
Phylogenetic analysis of *Culicoides sonorensis* D-7 like proteins. A total of 24 long and short form variant D7 protein sequences from *Culicoides, Culicinae, and Anopheline* species were aligned and a maximum likelihood unrooted tree generated using MEGA5 software based on the JTT matrix based model.

In *C. sonorensis,* nine proteins, each with a single PBP-GOBP domain, were identified in the secretome. A ClustalW alignment of these proteins with the short form variant d7r4 from *A. gambiae* showed conservation of the requisite cysteine residues, but amino acid identity between the sequences was low (<40%) suggesting these proteins are functionally diverse (Fig. 6). All the full length *C. sonorensis* D7 proteins appear to be short form variants with molecular masses <20 kDa and a single OBP domain. The two D7 proteins with partial sequences reported also only have a single OBP domain, but they cannot be conclusively identified as short form variants until their entire sequence is determined. A comparison of *Culicoides* D7 proteins with their Ceratopogonidae or Psychodidae counterparts shows these proteins are, at best, distantly related (Fig. 7).

## Conclusions

This study expands our understanding of proteins produced in *Culicoides* saliva and emphasizes the limitations inherent in predicting protein function based on sequence similarity alone. Due to the limited sequence orthology between *Culicoides* and other Dipterans, as exemplified by the ‘unknown’ and ‘novel’ proteins in the secretome, more extensive characterization will be required particularly when it comes to determining function as it relates to feeding activity. To this end, future work with recombinant protein expression of salivary proteins may allow for functional classification and aid in the identification of the secretome factors that may be important in the successful transmission of arboviruses such as BTV, EHDV, VSV, SBV, and AHSV to susceptible hosts.
